# Does Topical Tretinoin Used for Chemoprevention Cause Increased Mortality?

**DOI:** 10.4103/0974-2077.58526

**Published:** 2009

**Authors:** Prabhakar M Sangolli

**Affiliations:** *Department of Dermatology, Dr. B. R. Ambedkar Medical College, Bangalore, Karnataka, India*

Retinoid compounds are known for their chemo-prop hylactic property in preventing common skin cancers, namely squamous cell carcinoma (SCC) and basal cell carcinoma (BCC).

Non melanoma skin cancers (NMSCs) account for one third of all malignancies in US. They cause enormous morbidity, economic burden, facial deformities and annual mortality of about 1000 patients. Prevention of these malignancies can therefore contribute to public health significantly.

Recently a study was conducted[1] which sought to investigate the role of topical tretinoin in prevention of skin cancers. During the course of the trial, the authors found an inexplicable, surprising, but significant finding; that patients who applied tretinoin had greater mortality. This lead to stoppage of the trial. This finding is of significance to practicing dermatologists and is being analyzed in this commentary.

## STUDY DETAILS

The basic objective of the study conducted by Weinstock *et al*., titled "Veterans Affairs Topical Tretinoin Chemoprevention (VATTC) Trial" was to determine whether application of high strength topical tretinoin (0.1%) will have chemo-prophylactic role in the prevention of common skin cancers.[1] Secondary objective was to determine its effect in terms of prevention of actinic keratoses and to stratify in whom this product could be useful.

## DESIGN

The trial was a blinded, randomized trial with 2-6 year follow up. The oversight was carried out by multiple independent committees. It was conducted at 6 medical centres of the US Department of Veterans Affairs located in Durham, North Carolina: Hines, Illinois: Miami, Florida: Long Beach, California: Oklahoma City, Oklahoma and Phoenix, Arizona.

## INCLUSION AND EXCLUSION CRITERIA

Patients with previous history of 2 keratinocyte carcinomas but not involving perianal or genital skin in the last 5 years and free from skin cancers at the time of randomization were included in the study.

Patients unable to give informed consent, non compliant patients, patients on PUVA therapy, patients treated previously with oral retinoids in the previous 6 months, patients with invasive muco-cutaneous carcinomas in the last 5 years, patients suffering from metastatic cutaneous cancers and patients with end stage cardiac disease were excluded from the study.

## MATERIALS AND METHODS

Patients applied tretinoin cream (0.1%) twice daily over entire face including ears for a period of 1 year. Recruitment period was from 1^st^ November 1998 up to 1^st^ November 2002. Patient follow up was done till 15^th^ November 2004. Data was analyzed by various statistical methods.

A total of 1131 patients were randomized. For tretinoin treatment group, 566 patients were recruited. The remaining patients received a placebo cream. Primary end point of the study was occurrence of new SCC or BCC. Monitoring of mortality was done.

## RESULTS

No adverse effects were seen in the tretinoin treated group. However, as many as 135 patients in the treatment group died during study period which was statistically significant. In view of this, Central Human Rights Committee asked investigators to stop intervention and continue follow-up of the patients only, till the completion of the study period. Vigorous efforts were made to analyze the cause of death such as quantity of the drug used by the patients. Vascular disorders were noted as the leading cause of death. Small-cell lung cancer, respiratory, cardiovascular and mediastinal diseases were the other conditions responsible for death of patients in the tretinoin group.

## COMMENT

The VAATC was a 6 year randomized chemoprevention study aimed at reducing the incidence of squamous cell carcinoma and basal cell carcinoma of the skin. Due to unexpected increased mortality in randomized group, trial had to be halted 6 months earlier than the scheduled period.

During the analysis of the possible causes of this mortality, some limitations in the study were noted: Use of multiple review boards instead of a single review board, absence of central oversight committee, lack of data on smoking habits of patients, inclusion of elderly population (who are exposed to many other confounding factors for mortality) and lack of data on some possible factors responsible for deaths.

The idea of topical tretinoin application as a cause of mortality appears implausible on theoretical considerations; several questions need to be answered to explain this finding. What could be the cause and mechanism? What could be the dosage of application which can explain this finding?

One possible cause of mortality could be respiratory complications especially in smokers, as reported in a previous study in patients on oral isotretinoin and β carotene.[2] Retinoic acid syndrome is another possible cause.[3] Retinoic acid syndrome which may be lethal, is characterized by fever, respiratory distress, serositis, pulmonary oedema and infiltration. It has been reported to occur after administration of all-trans retinoic acid (ATRA) most commonly in patients with acute promyelocytic leukaemia (APL). However percutaneous absorption of tretinoin which varies between 1% and 8% is insufficient to cause lethal respiratory complications that are seen in retinoic acid syndrome. Advanced age of patients (which was not included as an exclusion criterion in the study) and high dose of tretinoin applications could be other factors which may have contributed to increased mortality in the study.

An editorial in the same issue urges readers to judge results with discretion till additional data is available. [4] The editorial urges practitioners to discuss these results with elderly patients. The editoial further recommends that prescribing physicians should inform all potential patients about the findings of the study. The editorial also suggested that measures should be taken to design studies with due attention given to risk factors like smoking. Further studies should also have foolproof stopping strategies backed by strong statistical methods to prevent premature termination of trials

Sir Osler's following words of wisdom are relevant even today as the present study also suggests.

"I would urge you to cultivate a keenly sceptical attitude toward the pharmacopoeia as a whole".
